# Supplementary material for: Scalable Nanopore sequencing of human genomes provides a comprehensive view of haplotype-resolved variation and methylation
Source: bioRxiv. 2023 Apr 5:2023.01.12.523790. Originally published 2023 Jan 15. Preprint. [Version 2] doi: 10.1101/2023.01.12.523790 (PMC9882142; doi:10.1101/2023.01.12.523790)
Supplement: Supplement 2 [file NIHPP2023.01.12.523790v2-supplement-2.pdf]

bioRxiv preprint doi: https://doi.org/10.1101/2023.01.12.523790; this version posted April 5, 2023. The copyright holder for this preprint (which was not certified by peer review) is the author/funder, who has granted bioRxiv a license to display the preprint in perpetuity. It is made available under aCC-BY 4.0 International license.

Supplementary Tables

(see attached Excel spreadsheets)

Supplementary Table 1. Information about the ONT sequencing runs and computational pipeline performance.

Supplementary Table 2. Additional information about benchmarking small variant calls.

Supplementary Table 3. Assembly statistics and benchmarks.

Supplementary table 4. Structural variant benchmarks

Supplementary table 5. Methylation analysis statistics

Supplementary table 6: Analysis of rare SVs in brain samples

bioRxiv preprint doi: https://doi.org/10.1101/2023.01.12.523790; this version posted April 5, 2023. The copyright holder for this preprint (which was not certified by peer review) is the author/funder, who has granted bioRxiv a license to display the preprint in perpetuity. It is made available under aCC-BY 4.0 International license.

Supplementary Figures

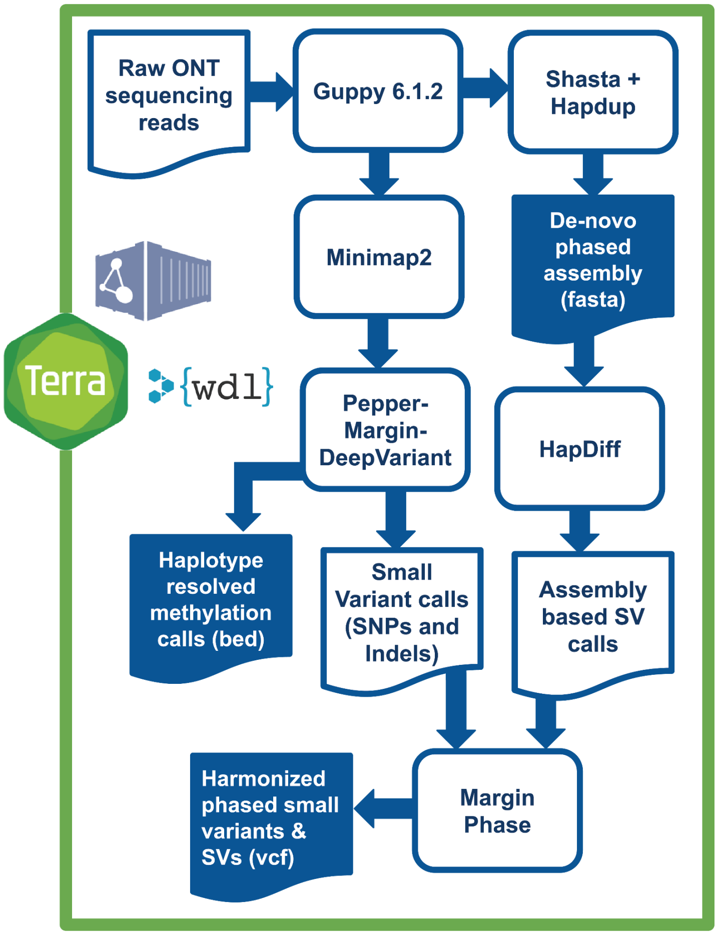

Supplementary Figure 1. Overview of variant calling and methylation analysis pipeline. Raw ONT sequencing reads are basecalled by Guppy 6.1.2, which simultaneously produces methylation tags. A diploid, de-novo phased assembly is produced using a combination of Shasta and HapDup. These assemblies are used to call structural variants with HapDiff. Small variants are called against a reference genome with Pepper-Margin-DeepVariant. The phased alignment file generated by Margin is used to produce haplotype-resolved methylation calls. Small and structural variants are jointly phased by Margin, producing a single harmonized vcf.

bioRxiv preprint doi: https://doi.org/10.1101/2023.01.12.523790; this version posted April 5, 2023. The copyright holder for this preprint (which was not certified by peer review) is the author/funder, who has granted bioRxiv a license to display the preprint in perpetuity. It is made available under aCC-BY 4.0 International license.

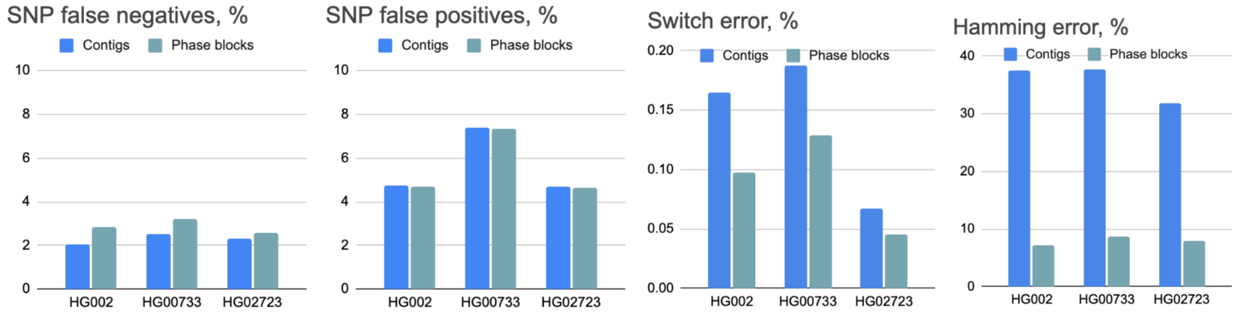

Supplementary Figure 2. Assemblies switch and hamming errors computed using “whatshap compare”. SNPs were called using dipcall and benchmarked against small variant calls in HPRC assemblies.

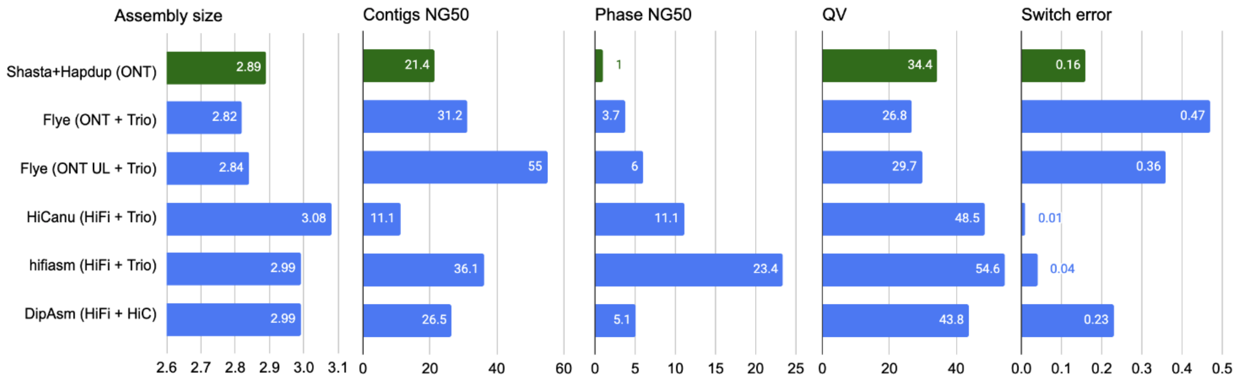

Supplementary Figure 3. Assembly metrics comparison against HG002 assemblies produced in Jarvis et. al (2022). Our assemblies are highlighted in green. Flye (ONT+Trio) were produced using standard ONT reads at 60x coverage and Illumina parental information; Flye (ONT UL + Trio) is similar, but using ultra-long ONT extraction. HiCanu and hifiasm used 34x HiFi reads and Illumina parental sequencing. DipAsm used 34x HiFi reads and 60x Hi-C reads. Original evaluations from Jarvis et al. are shown. See Supplementary Table 3 for more detail.

bioRxiv preprint doi: https://doi.org/10.1101/2023.01.12.523790; this version posted April 5, 2023. The copyright holder for this preprint (which was not certified by peer review) is the author/funder, who has granted bioRxiv a license to display the preprint in perpetuity. It is made available under aCC-BY 4.0 International license.

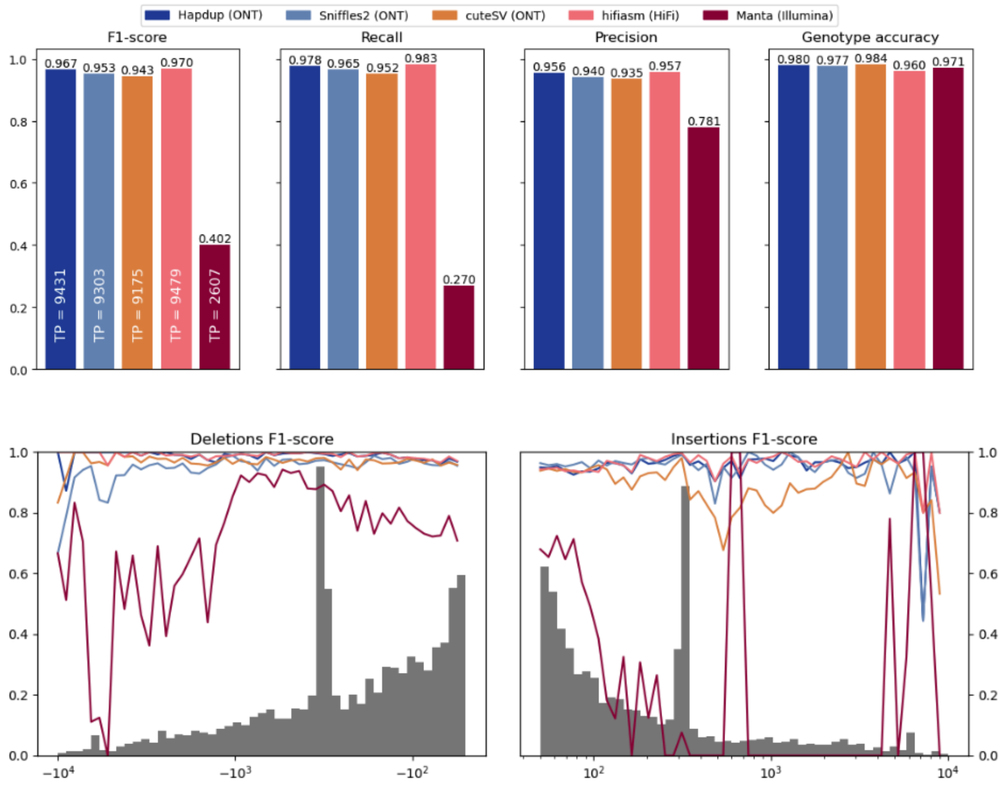

Supplementary Figure 4. Comparison of structural variants using GIAB HG002 benchmark, including Illumina-based calls produced with Manta.

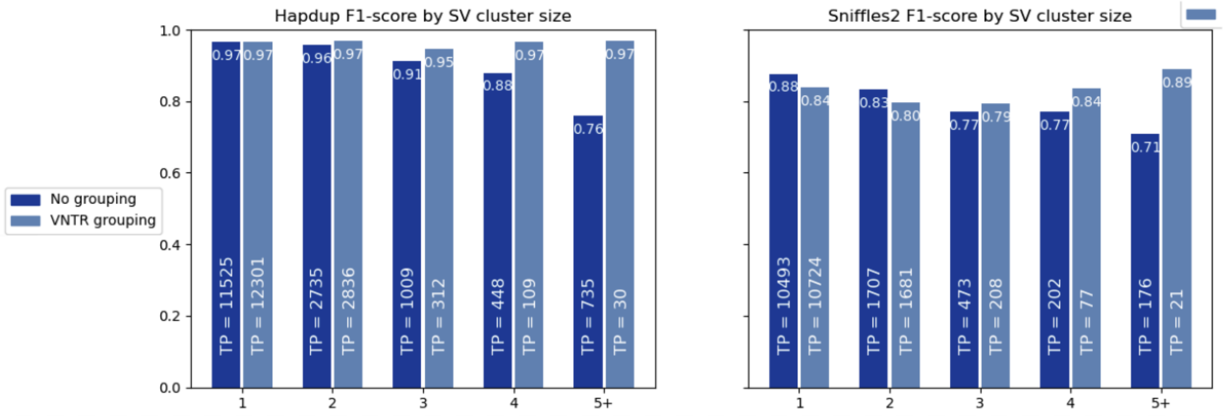

Supplementary Figure 5. F1-score for SV inside clusters of different sizes. The HiFi calls for HG002 genome were used as reference, and calls within 2 kbp were clustered using single linkage clustering. The number of true positive calls in each category is shown as text. When VNTR grouping is enabled, all insertions and deletions within the same haplotype in a single VNTR are combined into a single call. A substantial portion of the reduced Sniffles2 concordance is explained by the differences in representation of SV clusters by the assembly-based and mapping-based approaches.

bioRxiv preprint doi: https://doi.org/10.1101/2023.01.12.523790; this version posted April 5, 2023. The copyright holder for this preprint (which was not certified by peer review) is the author/funder, who has granted bioRxiv a license to display the preprint in perpetuity. It is made available under aCC-BY 4.0 International license.

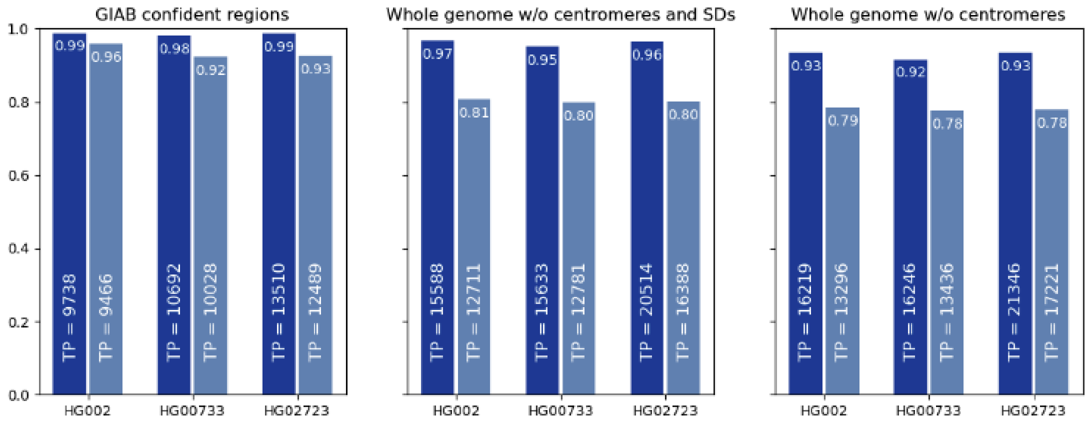

Supplementary Figure 6. Comparison of Hapdup and Sniffles2 F1-scores against HiFi assemblies.

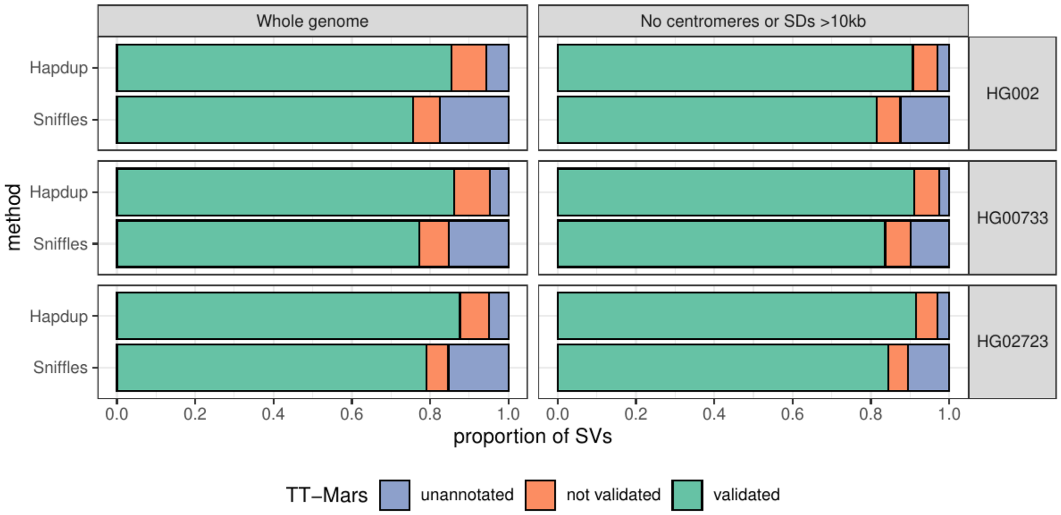

Supplementary Figure 7. TT-Mars evaluation of Hapdup and Sniffles2 calls. Structural variant calls from Hapdup and Sniffles2 were compared to the assemblies from the HPRC for HG002 (top), HG00733 (middle), and HG02723 (bottom) with TT-Mars. The calls were either validated by the alignment (green), not validated (orange), or couldn't be annotated by TT-Mars (blue). We evaluated all SVs across the genome (left), as well as the subset of SVs that don't overlap centromeres or segmental duplications larger than 10 Kbp (right).

bioRxiv preprint doi: https://doi.org/10.1101/2023.01.12.523790; this version posted April 5, 2023. The copyright holder for this preprint (which was not certified by peer review) is the author/funder, who has granted bioRxiv a license to display the preprint in perpetuity. It is made available under aCC-BY 4.0 International license.

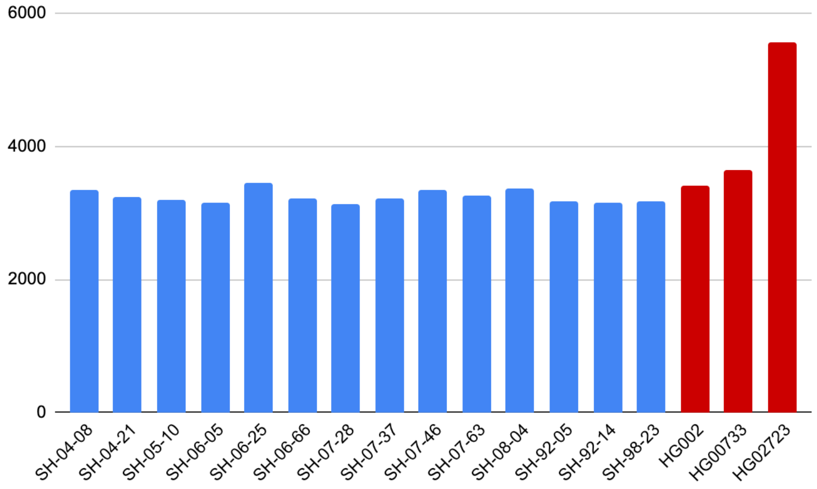

Supplementary Figure 8. Number of SVs inside clusters of size at least 2. SVs are clustered with single linkage clustering within 2kb. Multiple indels within a single VNTR element are considered as single SVs in this analysis.

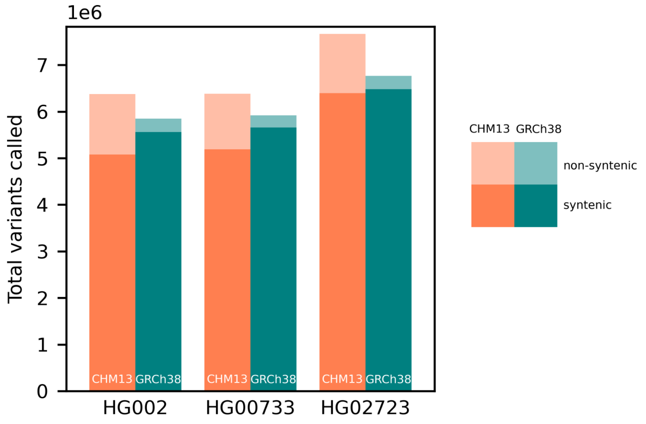

Supplementary Figure 9. Comparison of small variant calling using GRCh38 and T2T-CHM13 references.

bioRxiv preprint doi: https://doi.org/10.1101/2023.01.12.523790; this version posted April 5, 2023. The copyright holder for this preprint (which was not certified by peer review) is the author/funder, who has granted bioRxiv a license to display the preprint in perpetuity. It is made available under aCC-BY 4.0 International license.

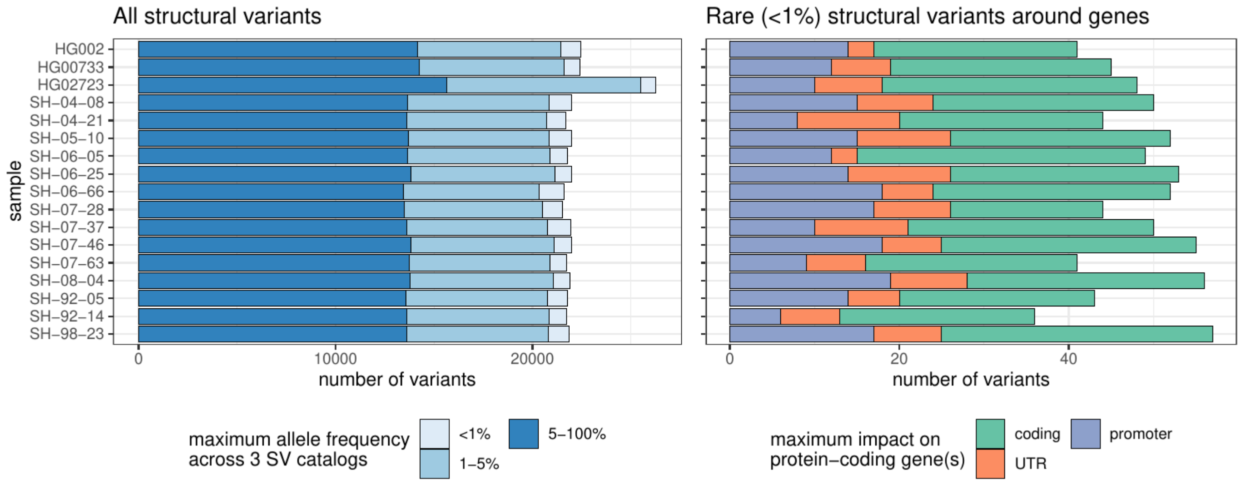

Supplementary Figure 10. Lenient structural variant catalog. Similar to Figure 7A but including SVs close to centromeres, telomeres, or within segmental duplications were removed. (A) Number of structural variants across samples. In the left panel, structural variants were annotated with three SV catalogs (the gnomAD-SV database, a long-read-based SV catalog, and the HPRC v1.0 SV catalog). SVs are matched if they have at least 10% genomic overlap. The colors highlight the maximum frequency across these catalogs, the lighter blue showing “rare” SVs (with an allele frequency below 1%) in the catalogs, or unmatched. SVs may be unmatched, either because they are novel or due to the difficulties in the database comparison. The right panel shows the number of rare structural variants in protein-coding genes, grouped by their impact on the gene structure.

bioRxiv preprint doi: https://doi.org/10.1101/2023.01.12.523790; this version posted April 5, 2023. The copyright holder for this preprint (which was not certified by peer review) is the author/funder, who has granted bioRxiv a license to display the preprint in perpetuity. It is made available under aCC-BY 4.0 International license.

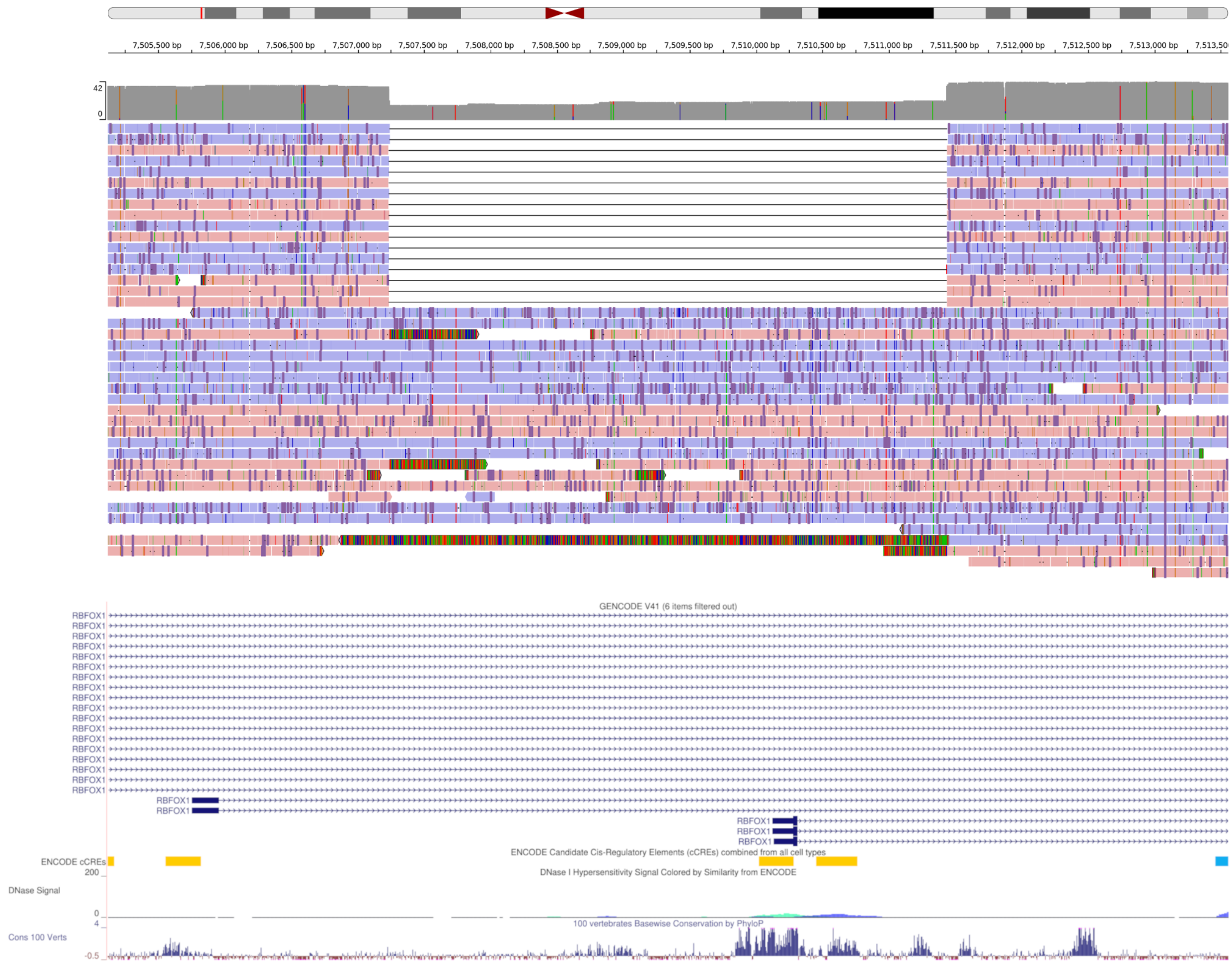

Supplementary Figure 11. IGV view of a 4.2 Kbp heterozygous deletion of a transcription start site and exon of *RBFOX1*. The coverage histogram (dark grey) shows the drop in read coverage. The alignment of about half of the reads, labelled by strand (red/blue), support the deletion. The GENCODE track, ENCODE candidate cis-regulatory elements, and conservation tracks are shown at the bottom.

bioRxiv preprint doi: https://doi.org/10.1101/2023.01.12.523790; this version posted April 5, 2023. The copyright holder for this preprint (which was not certified by peer review) is the author/funder, who has granted bioRxiv a license to display the preprint in perpetuity. It is made available under aCC-BY 4.0 International license.

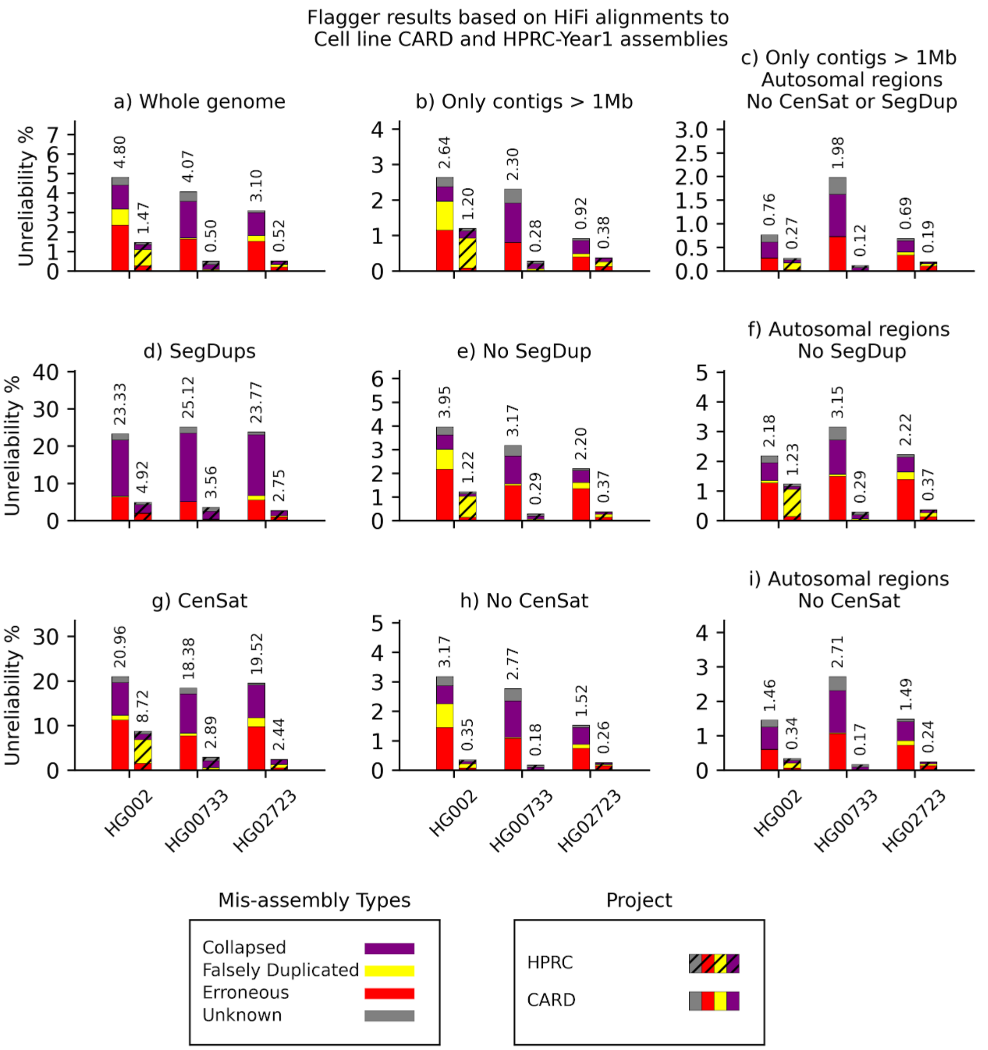

Supplementary Figure 12. Flagger results based on HiFi alignments to cell line CARD and HPRC-Y1 assemblies. The y-axis of each panel indicates the unreliability percentages which are the total number of bases flagged as misassembly divided by the total assembly length and multiplied by one hundred

bioRxiv preprint doi: https://doi.org/10.1101/2023.01.12.523790; this version posted April 5, 2023. The copyright holder for this preprint (which was not certified by peer review) is the author/funder, who has granted bioRxiv a license to display the preprint in perpetuity. It is made available under aCC-BY 4.0 International license.

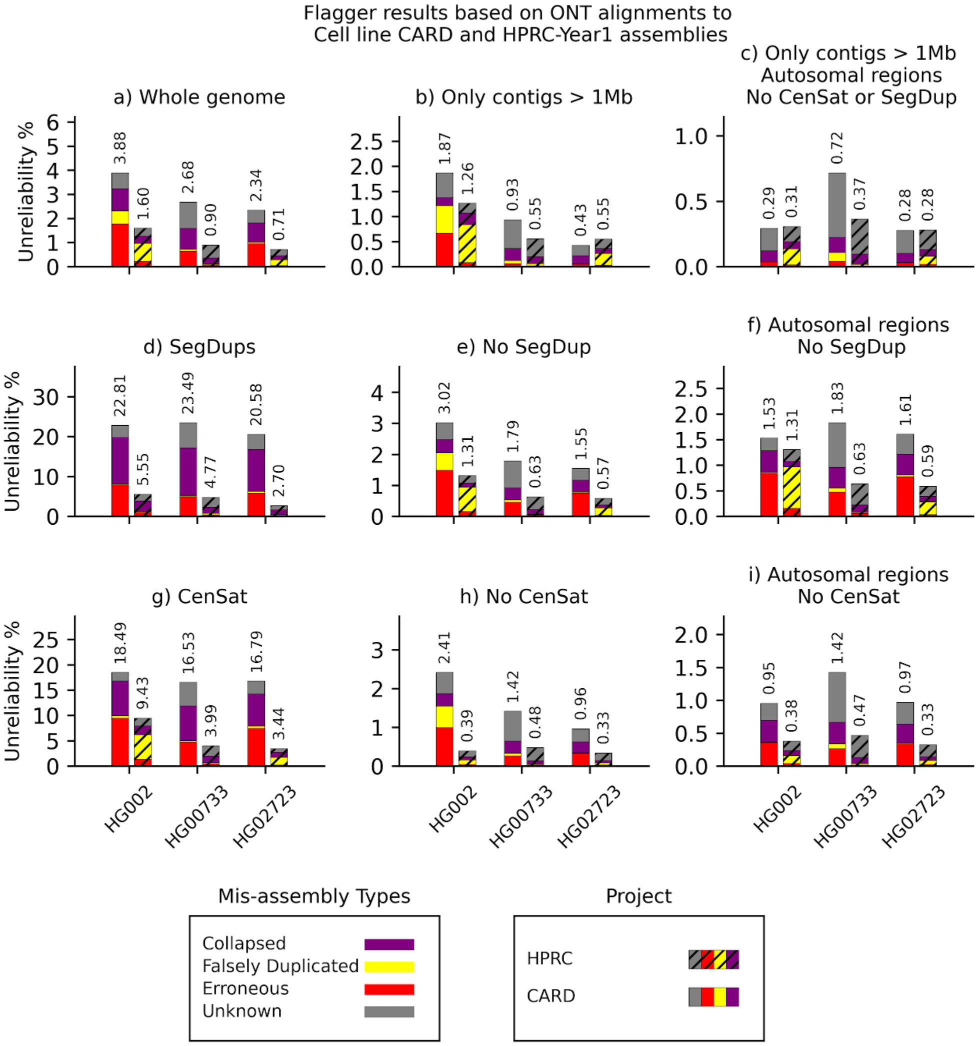

Supplementary Figure 13. Flagger results based on ONT alignments to cell line CARD and HPRC-Y1 assemblies. The y-axis of each panel indicates the unreliability percentages which are the total number of bases flagged as misassembly divided by the total assembly length and multiplied by one hundred.

bioRxiv preprint doi: https://doi.org/10.1101/2023.01.12.523790; this version posted April 5, 2023. The copyright holder for this preprint (which was not certified by peer review) is the author/funder, who has granted bioRxiv a license to display the preprint in perpetuity. It is made available under aCC-BY 4.0 International license.

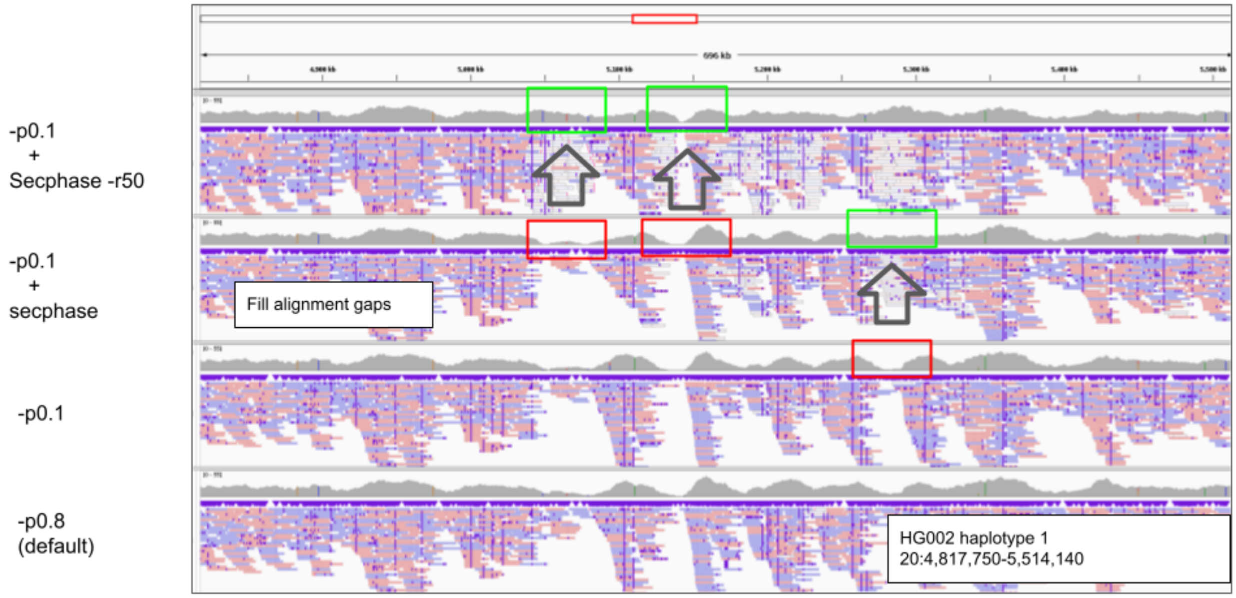

Supplementary Figure 14. Adopting Flagger to ONT reads. Changing -p0.8 to -p0.1 increased the number of secondary alignments and filled some alignment gaps. Secphase with -r50 could not fill more of the alignment gaps with secondary alignments. The parameter -r50 was added to randomly assign reads in highly homozygous regions.

bioRxiv preprint doi: https://doi.org/10.1101/2023.01.12.523790; this version posted April 5, 2023. The copyright holder for this preprint (which was not certified by peer review) is the author/funder, who has granted bioRxiv a license to display the preprint in perpetuity. It is made available under aCC-BY 4.0 International license.

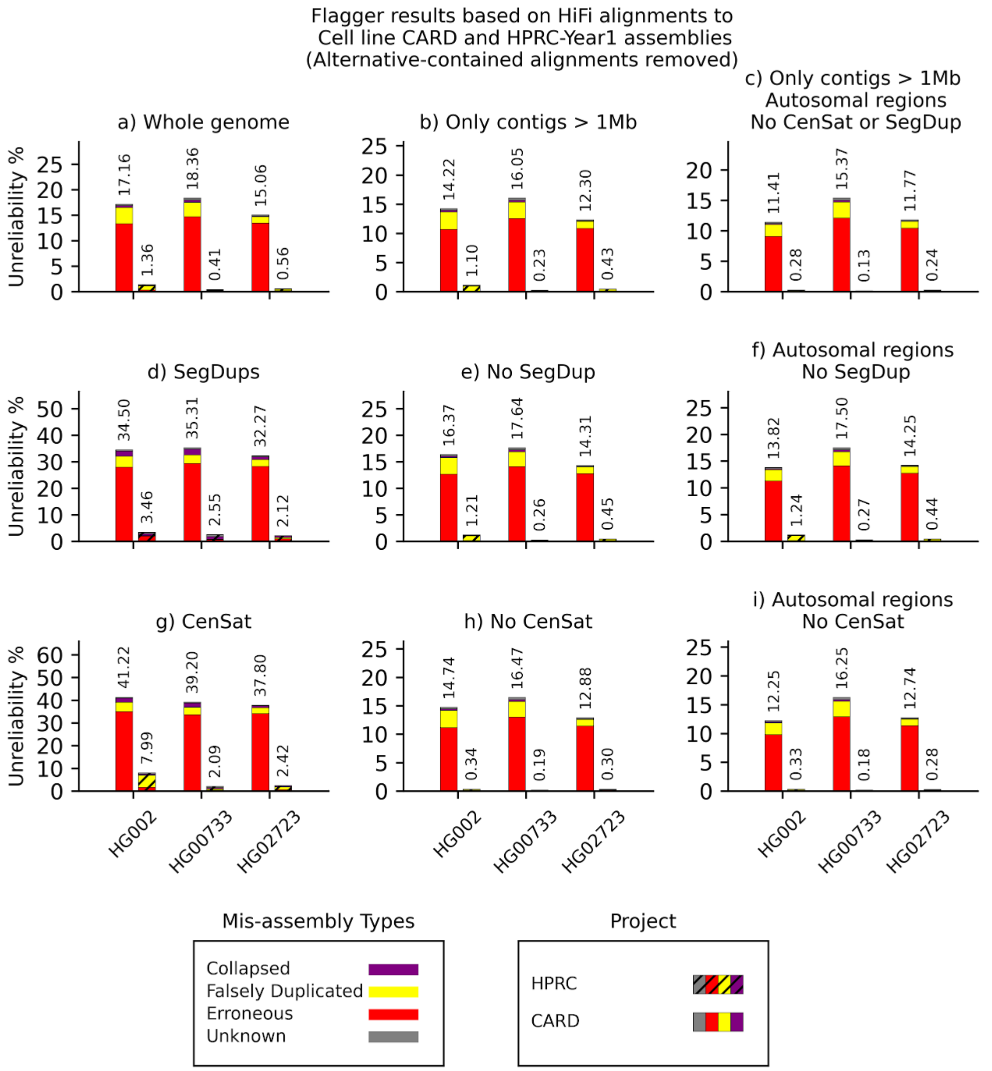

Supplementary Figure 15. Flagger results based on ONT alignments to cell line CARD and HPRC-Y1 assemblies after removing alternative-contained alignments. The y axis of each panel indicates the unreliability percentages which are the total number of bases flagged as misassembly divided by the total assembly length and multiplied by one hundred
